# An Epitome of Juridical or Forensic Medicine; for the Use of Medical Men, Coroners, and Barristers

**Published:** 1817-01

**Authors:** 


*An Epitome of Juridical or Forensic Medicine; for the Use of Medical Men, Coroners, and Barristers.* By George Edward Male, M. D. One of the Physicians to the General Hospital in Birmingham. 8vo. pp. 199. London, 1816.

Medical Jurisprudence includes two distinct branches: Juridical Medicine and Medical Police. The objects of both are alike dignified and comprehensive. They regard the preservation and welfare of civilized man. Medical Police is principally occupied in exploring the means whereby the public health may be preserved and population encouraged, or in illustrating their application. Juridical Medicine consists in applying the precepts and experience of medicine and the accessory sciences to the solution of dubious questions in the practice of legislation. Hence it embraces the conditions of health and derangement in the human œconomy, the causes and phænomena of injury and death. In fact, all the functions of mind and body, and their lesions, physically considered, come within the sphere of its cognizance. The character, the life, and fortune of individuals, are frequently implicated in its decisions.

In England, medicine and its professors have gradually risen to an elevation in rank and dignity, which they have no where else acquired. The English character is signalized by its love of justice—by the jealousy and circumspection with which every thing, that even remotely interests the life and liberty of the subject is regarded. That by a nation, thus liberal and independent, a branch of science, calculated so powerfully to controul the disorders of the social system, to rescue innocence from infamy and suspicion, and lead to the exposition and punishment of guilt, should have been so long imperfectly appreciated and applied, is a problem, which nothing but a familiar acquaintance with the natural inconsistencies of the human character would have enabled us to resolve. It constitutes one of those blemishes of the national legislation, which, however painful the survey, or faint the prospect of remedy, cannot too strongly fix the attention of the philosopher. If his efforts to trace the evil to its source be unavailing, he will, at least, acquire correct notions of its magnitude: he may expose the malignity of its operation, although he fail to arrest its progress. But the time, we verily believe, is drawing nigh when the connection, which subsists between medicine and legislation, will be more clearly discerned among us; when the knowledge of the juridical physician and surgeon will be more confidently sought for and consulted; and its application be allowed to exercise a deeper influence on the researches of the coroner and the decisions of the bar. In this view, we cannot but hail as auspicious the appearance of the volume, which stands announced at the head of this article. We would fain regard it as a gleam of day breaking in upon the obscurity, in which, to the disgrace of a politic and enlightened people, we have so long suffered juridical medicine to be involved.

Medicine and Jurisprudence, as necessarily resulting from the wants, the infirmities, and profligacy of man, must have been nearly coeval with his origin. Like the diseases and crimes which they were instituted to remedy and repress, these sciences, if such we may then call them, long retained a rude and simple character. Theirs, like that of the other sciences, was an infancy of ages. They sprang up wholly unconnected. No trace of the relations which, at a remote period of their developement, would serve to connect them, or of the stability and light reciprocally afforded by those relations, was then perceptible. But as the human mind emerged from primæval darkness and simplicity; as its pursuits were diversified, and population advanced, the constitution of society became gradually more obnoxious to physical and moral disorders. Disease and profligacy kept pace with the progress of civilization. Infection was generated. Crimes assumed a character more atrocious and complicated. An alliance more intimate was formed between artifice and guilt. It was then that the legislator first had recourse to the experience of the physician. By it, he was frequently enabled to avert the scourge of contagion from the city or the camp, to arrest its desolating stride; or, perchance, to solve some doubtful problem which came before him in his judicial capacity. Thus the knowledge of philosophers and physicians, imperfect as it then must have been, was applied to subjects of legal and political research: and from the relations of medicine with law, Medical Jurisprudence derived its origin.

In the writings of the great law-giver of Israel, (1) we first clearly descry the rise of the infant science. Vestiges of its early influence on the legislation of Egypt and Greece (2) are also perceptible. But during the more peaceful and flourishing periods of the Roman empire, are we principally enabled to trace its progress. At an early period, orders were issued to inspect the internal organs of women dying in pregnancy: and the bodies of the murdered were publicly examined, with the view of facilitating the discovery of the delinquent. Under the beneficent reigns of Severus and Antoninus, the law was repealed, by which the crime of procured abortion, during every stage of utero-gestation, had been before indiscriminately punished with death. (3) Upon these examples of the application of medicine to the system of jurisprudence, among the Romans, many others, if it were necessary, might be accumulated. And wherever, from the extension of her conquests, the laws of Rome were received, it was customary to take the opinion of philosophers and physicians in all extraordinary cases.

But little interest will be derived from a contemplation of juridical medicine at this remote æra; when an acquaintance with the structure, physiology, and lesions of the human frame, which could alone give stability to its character and researches, was precluded by insuperable prejudices; when its practice was debased by superstitions the most deadly and revolting. In fact, from the institution of the code of Charles V. Emperor of Ger-

---

(1) *See* the books of *Genesis*, *Exodus*, and *Deuteronomy*.
(2) See *Diodorus of Sicily*.
(3) *Plutarch*, *Suetonius*, *Tacitus*.

many, about the middle of the sixteenth century, juridical medicine may be first said to have assumed a regular aspect, and to date its commencement as a science. By this code, the visit and report of physicians and surgeons were required in all criminal cases. We shall, therefore, content ourselves with a rapid survey of the progress which the science has made from that period to the present, in some of the more distinguished nations of Europe: and to enhance the interest and utility of this historical sketch, we shall enumerate a few of those authors by whose researches medical jurisprudence has been principally enriched and promoted. Our limits will not allow us to enter into an analysis of the different works, or examine by the torch of criticism the opinions which they respectively exhibit. We shall, indeed, do little more than announce their titles, under the head of the country in which they were published, and, as nearly as possible, in the order of their publication.

In *Germany,* state-medicine *(staatsarzneikunde),* as it is there not inappropriately termed, has been long cultivated with unparallelled ardour and success. The peculiar constitution of the German character, and the minute distinctions introduced into the German system of criminal legislation, probably both incited her medical philosophers to this path of research, and signally favoured the exertion of their talents. The honour of having produced the earliest writings on this subject belongs exclusively to them. In the *Constitutio Criminalis Carolina,* published about the year 1545, the rudiments of juridical medicine were first clearly developed. This was succeeded by the productions of *Struppe, Boerner,* and *Kannegiesser.* But we are utterly unacquainted with the contents, or even the title, of any German work by which medical jurisprudence has been illustrated, anteriorly to the close of the seventeenth century. A mere enumeration of the writings which have subsequently been published upon the science and its different branches, would occupy a space far more considerable than our limits will enable us to devote to the whole article. We shall almost exclusively restrict ourselves to the notice of those authors who have written systems of state-medicine, or valuable collections upon the subjects immediately connected with it. For an account of the best monographs which have appeared on various branches of the science, we refer the reader to the series of articles some time since commenced in the Selection Department of this Journal.

About the commencement of the eighteenth century, Michaelis Valentini (1) published, at Frankfort, his celebrated Pandects. They form a complete collection of the decisions and opinions of different juridical physicians who had preceded him. This work was followed by those of Zittman, (2) Alberti, (3) Richter, (4) and Teichmeyer. (5) Unable to procure these from our bookseller, we can say nothing of their respective merits; nor can we precisely indicate the period of their publication. In 1730, and previously, if we err not, to the four last mentioned, Starck (6) published his work on the utility of medicine in jurisprudence. Some years afterwards appeared, at Leipsic, the publication of Hebenstreit; (7) and subsequently, though we are unacquainted with their exact dates, the Institutions of Ludwig, (8) and the Elements of Fazellius. (9) Plenck, (10) in 1781, gave to the world his useful elementary book; and, in the following year, the first volume of Haller's (11) Lectures on Juridical Medicine came out. The work was shortly after completed in three volumes. The year 1792 gave birth to the Conspectus of Sikora, (12) which, within a short period, was succeeded by the First Lines of Loder, (13) the excellent System of Metzger, (14) and the Delineations of Muller. (15) Our historical sketch of the medico-juridical literature of the eighteenth century closes with the voluminous collection published by Schleget. (16)

---

(1) Pandectæ Medico-legales. 4to. Francofurti, 1702.

(2) Zittman, Medicina Forensis. 4to Francofurti.

(3) Systema Jurisprudentiæ Medico-legalis, tom. vi. 4to.

(4) Decisiones Medico-forenses.

(5) Institutiones Medicinæ-legales. 4to. Jenæ (edition by Fazellius).

(6) De Medicinæ utilitate in Jurisprudentia. 4to. Helmstadii, 1730.

(7) Anthropologia Forensis. 8vo. Lipsiæ, 1753.

(8) Institutiones Medicinæ Forensis.

(9) Elementa Medicinæ Forensis.

(10) Elementa Medicinæ et Chirurgiæ Forensis. 8vo. Viennæ, 1781.

(11) Vorlesungen über die gerichtliche Arzneywissenschaft. 3 B. 8vo. Bern, 1782-4.

(12) Conspectus Medicinæ-legalis. Pragæ et Dresdæ, 1792.

(13) Anfangsgründe der Medicinischen Anthropologie und der staatsarzneykunde. 8vo. Weim, 1793.

(14) System der gerichtlichen arzneywissenschaft. 8vo. Konigsb. 1793.

(15) Entwurf der gerichtlichen arzneywissenchaft. 2 B. 8vo. Frankf. 1796-8.

(16) Collectio opusculorum selectorum ad Medicinam-forensem spectantium: Curante F. Ch. Tr. Schleget. Tom. viii. 8vo. Lipsiæ, 1789, (1800).

It includes a period of several years. The last volume, we believe, appeared in 1800. During this century, a few works, exclusively devoted to the subject of medical police, were produced. Those which have principally come within our knowledge are, the diffusive System of Frank, (1) and the more concise productions of Hüsty (2) and Hebenstreit. (3)

The nineteenth century, if we may be allowed to draw an inference from the bibliographical departments of the Foreign Journals, has not been less prolific than its predecessor in German writings on state-medicine. Peace, contrary to our hopes and expectations, has hitherto done little towards facilitating the literary intercourse between this country and the more distant parts of the European continent. The utmost difficulty in procuring the later medical publications of Germany continues to be experienced. Hence our knowledge of her literature in this branch of the science, during the present century, is extremely confined and superficial; and our sketch of it must necessarily be slight and imperfect. Metzger, (4) in 1804, published another work upon juridical medicine, consisting of two volumes. About two years afterwards, Knape and Hecker commenced, at Berlin, a periodical publication, under the title of Critical Annals of Political Medicine. (5) We know not whether it is yet continued. In the year 1808, a new work, upon a somewhat similar plan, entitled Annals of State Medicine, was announced and published by the celebrated Professor Kopp, (6) of Hanau. It has regularly appeared in the succeeding years; and is, we believe, yet going on. The few productions subsequent to this period, of which we have been able to gain any knowledge, are the systematic Manual of Bernt, (7) and the more limited treatises and discus-

(1) System einer Medicinischen Polizey. 12 B. 8vo. Frankf. 1794.

(2) Über die Medicinische Polizey. 2 B 8vo. Presb. 1786.

(3) Lehrsätze der Medicinischen Polizeywissenschaft. 8vo. Leipsig, 1791.

(4) Gerichilich Medic. Abhandlungen. 2 B Konigsb. 1804.

(5) Kr[illegible]tische Jahrbuecher, &c. Von Chr. Knape und Aug. Fr. Hecker. Berlin, 1806.

(6) Jahrbuch der Staatsarzneikunde, herausgegeben von Dr. J. H. Kopp, Hofrath und Professor [illegible]n Hanau Frankfurt am Main.

(7) Systematisches H[illegible]ndbuch der gerichtlichen Arzneikunde, zum gebrauch fur Aerzte, Wundarzte, Rechtsgelehrte und zum Leitfaden fur offentliche vorlesungen. 8vo. Prag. 1813.

sions upon the science, by Hochweis, (1) Joerg, (2) Platner, (3) and Henke. (4) — Platner, (5) Fischer, (6) Stoll, (7) Ludwig, (8) Bruckner, (9) Ristelhuber, (10) and Josephi, (11) are some of the principal German authors who have written on medical police, from the commencement of the present century.

*Italy,* in her attention to medical jurisprudence, has emulated the example of her neighbours. Even about the middle of the 17th century, physicians were there consulted in criminal cases. But the earlier writers, tainted by the prevalent spirit of their age, seem to have cultivated the science, rather as casuists than as philosophers. The productions of Fortunatus Fidelis, (12) Ammannus, and others, which have descended to us on the stream of time, certainly exhibit much less of sound medicine than of theological controversy. Among these, the work of Paul Zacchias, (13) although unnecessarily verbose, voluminous, and clogged with ecclesiastical discussions foreign to the purpose, stands pre-eminent. In the composition of it, he has extracted every thing of value from the writings of his predecessors; and it is enriched with all the knowledge of his age in medicine and legislation. About the

---

(1) Anleitung zur Abfassung gerichtlicher Untersuchungsberichte. Gratz, 1814.

(2) Taschenbuch fur gerichtliche Aerzte und Geburtshelfer, &c. 8vo. Leipzig, 1814.

(3) Quæstiones Medicinæ Forensis, XLI. Deprecatio pro crimine infanticidii. Lipsiæ, 1814.

(4) Abhandlungen aus dem Gebiete der gerichtlichen Medicin, &c. 1er. Band. 8vo. 1815.

(5) Quæstiones Medicinæ Forensis, XLII. Publicæ curandæ valetudinis præsidia in civitate jure pleno desiderari ostenditur. 4to. Lipsiæ, 1814.

(6) Darstellung der Medicinalverfassung Sachsens, &c. 8vo. Leipzig, 1814.

(7) Staatswirthschaftliche Untersuchungen u, Erfahrungen uber das Medicinalwesen nach seiner Verfassung, Gesetzgebung und Verwaltung, &c. Zurich, 1814.

(8) Progr. de damno et calamitate, quæ insanitatem publicam et societatem ex perpetuo bello redundant. Pars i. ii. Lipsiæ, 1814.

(9) Ueber Einrichtung und Verpflegung stehender Feldspitaler, &c. 8vo. Leipzig.

(10) Versuch uber den Militeur-Hospitaldienst im Allgemeinen, &c. 8vo. Cassel, 1814.

(11) Anweisung zur Erhaltung der Gesundheit der Soldaten im Felde. Rostock, 1814.

(12) De Relationibus Medicorum.

(13) Quæstiones Medico-Legales. Tom. ix. 4to. Romæ, 1621.

middle of the eighteenth century, appeared a publication from the pen of the celebrated Beccaria. (2) The Institutions of the Italian Professor (3) must have been the production of a much less remote period. All our efforts to procure it, or ascertain the place and date of its publication, have been unavailing.

From the time of Francis I. the application of medicine to jurisprudence by the *French,* may be properly dated. The necessity of having recourse to physicians in many cases upon which the judge, from his own knowledge, was incompetent to decide, became obvious after the publication of the criminal code of Charles. And by the decrees of the kings of France, issued subsequently to that of the German Emperor, what had previously been practised but as custom, was transformed into a law. At that period, physicians and surgeons were indiscriminately employed in framing juridical reports. But Henry IV. in 1606, by letters patent, conferred on his first physician the privilege of nominating surgeons, in every town, to the exclusive exercise of this function. And Louis XIV. in 1667, formally declared all reports invalid, which had not received the sanction of at least one of the surgeons appointed by his principal physician. Yet, from this institution resulted few of the advantages which it was calculated to insure. Unqualified persons were allowed to purchase the appointment. These abuses, united to the jealousy which had long subsisted between the corporations of surgery and the colleges of medicine, gave rise, in 1692, to a new act of legislature. By this, physicians and surgeons were invested with the juridical office in common, on the payment of a stipulated sum. But surgeons, in those times, were not distinguished by the erudition which they now possess. Hence, in every thing which did not directly involve surgical discussions and practice, their reports were frequently defective. Magistrates, consequently, were induced to summon the more learned physician to the assistance of the juridical surgeon; and the practice, like many others, acquired force and regularity from repetition. *Ambrose Paré* was the first among the French who wrote expressly on juridical medicine. His treatise on reports, published in 1575, although infected by the prejudices of the age, bespeaks

---

(2) Scriptura Medico-Legalis, 1749.

(3) Instituzioni de Medicina Forense, di Guiseppe Tortosa, Professore Medico.

considerable sagacity; and was long employed by French surgeons as their only guide in these researches. At length, after the lapse of almost a century (1650), a treatise on the same subject appeared from the pen of *Gendri*, of Angers; and was succeeded by those of *Blegni*, Lyons, 1684; and of *Deveaux*, (1) Paris, 1693. The latter, which we have not seen, is represented as a meritorious production, and particularly excellent in whatever regards the diagnosis and prognosis of wounds.

The eighteenth century, memorable for the progress of the human mind, and for its conversion from the enthusiasm of poetry, and the fine arts, to the study of the sciences, was most auspicious to the culture of juridical medicine in France. The noble spirit of emulation, by which the Academy of Surgery, and Royal Society of Physicians, were actuated, produced men who reflected light upon every department of medical science. Professor *Louis*, secretary of the academy, taught publicly, in the schools, the art of resolving different questions in medical jurisprudence, till then unpractised. Numerous memoirs on its various branches successively appeared: and eloquence allied itself to knowledge in this novel mode of conferring benefits on mankind. Upon the great principles of justice and humanity which presided at the reform of the penal code, professorships of medical jurisprudence were created in all the faculties of medicine. This important object in the system of public instruction was accomplished by the law of 1792; and the law of 1800 declares all juridical reports void, which are not executed by a doctor of one of the faculties of medicine. Thus the science, in its advance, has kept pace with the progress of the human mind; and, from the emulation consequently excited in France, more books have, within a few years, appeared upon the subject, than had previously been produced during the lapse of many centuries. *Louis* published, at Paris, in 1788, his letters on the certainty of the signs of death, in answer to the dissertations of *Winslow* and *Bruhier*, together with his memoirs on drowning, and on the means of distinguishing suicide from assassination, in cases of death by suspension. His consultations on the celebrated causes of Monbailly, Calas, and Cassagneux, (2) remain monuments of his ex-

(1) L'art de faire les rapports en Chirurgie.

(2) *See* Recueil des causes célèbres.

alted talent. The question of the Cæsarean operation, in all its bearings, moral, political, and religious, was also discussed, with the hand of a master, by *Winslow*. *Petit*, *Bouvart*, and others, disputed the opinion of Louis upon protracted pregnancy. By the former, several memoirs were produced on the phænomena of hanging and strangulation. He had, likewise, occasion to investigate the question relative to the signs of death from abstinence. Salin examined the difference between the effects of corrosive sublimate and arsenic upon the animal organs; while Lafosse sought to distinguish the phænomena produced by death, from the traces of violence inflicted during life, upon the body. He, moreover, clearly developed the signs of pregnancy and parturition. In December 1789, Professor Chaussier demonstrated, in an excellent memoir, to the Academy of Sciences at Dijon, the importance of the study of juridical medicine. About this time, the Encyclopœdia (1) was undertaken; and Professor Mahon was one of the writers of the articles upon medical jurisprudence, contained in that great national work. The first systematic production on the science was published by Foderé, (2) in 1796.

During the few years of the present century, which have already elapsed, France has given birth to several valuable works on medical jurisprudence. Vigné, (3) Physician of Rouen, published, in 1805, his enlightened and humane reflections on the practice of the science. The small but useful work of Belloc, (4) and the first edition of the excellent system of Professor Mahon (5) appeared nearly together, in 1807. During the next year, the celebrated Marc (6) introduced in a French dress, the manual of juridical dissection, originally written in German by Roose; and augmented it with valuable compositions of his own on the pulmonary proof, and drowning. The same writer undertook for the French Dictionary of Medicine, (7) a series of articles on medical jurispru-

---

(1) Encyclopédie Méthodique.

(2) Les lois éclairées par les Sciences Physiques; ou Traité de Médecine légale et d'Hygiène Publique. Tom iii. 8vo. Paris.

(3) De la Médecine légale, &c.

(4) Cours de Médecine legale Theoretique et Pratique. 12mo. (An edition of this work has subsequently appeared in octavo).

(5) Médecine legale et Police Medical, &c. avec quelques Notes de M. Fautret, &c. Tom. iii. 8vo. Paris, last edition 1811.

(6) Manual d'autopsie cadavérique Medico legale, &c. 8vo. Paris

(7) Dictionaire des Sciences Médicales. Par une Société de Medicins et de Chirurgiens.

dence, such as, when completed, should form a comprehensive system of doctrine and practice in every branch of the science. This Colossal work, which will consist of at least sixty large octavo volumes, was commenced in 1812. Fifteen volumes only have yet reached us: and, hitherto, M. Marc has executed his engagement with considerable energy and talent. The year 1813 was distinguished by the appearance of the voluminous treatise of Foderé,(1) far outstripping its predecessors, as well in execution as in extent. Prunelle,(2) in 1814, published a discourse which had been delivered to the Faculty of Medicine at Montpellier, on the origin, progress, and practical application of the science: and, still more recently, a Selection of Memoirs, Consultations, and Reports, on different subjects of Medical Jurisprudence, was announed by Professor Chaussier.(3) We are not aware that the work has hitherto appeared in England. Independently of these productions, the French Medical Journals(4) abound with observations and reports in juridical medicine. We are not acquainted with any distinct system of medical police in the French language.

The reputable *work of Professor Mahon* was not published during his life-time. M. Fautrel undertook the charge of arranging the manuscript, illustrating it with notes, and giving it to the world. It consists of three volumes, and constitutes a system of doctrine both in juridical medicine and medical police. The first volume contains a biographical sketch of the Professor; a preface by the Editor; an introductory section, entitled *Généralités,* in which the origin and progress of juridical medicine are briefly traced, and the qualifications requisite for the practitioner, specified: and the following subjects are then discussed under distinct heads or sections. Impotence; sexual intercourse; castration; hermaphrodites; defloration; rape; sodomy; pregnancy; procrastinated

---

(1) Traité de Médecine légale et d'Hygiène Publique, ou de Police de Santé, adapté aux Codes de l'Empire Français et aux Connaissances Actuelles, &c. Tom. vi. 8vo. Paris.

(2) De la Médecine Politique en general, et de son objet; de la Médecine légale en particulier, de son origine, de ses progrès, &c. 4to. Montpellier, 1814.

(3) Recueil de Mémoires, Consultations et Rapports sur divers objets de Médecine légale.

(4) Particularly the excellent Journal de Médecine, by Leroux; the Bibliotheque Medicale, by Royer-Collard; and the Gazette de Santé, by Montegre.

birth; illegitimate birth; abortion; monsters; moles; doubtful conditions of mind and body; insanity; pretended, concealed, and imputed diseases. In the second volume are, successively treated, wounds; apparent death; violent death; examination of bodies; poisoning; and infanticide. The third volume terminates the department of juridical medicine, by articles on drowning; hanging; and juridical reports and consultations: and comprehends, under that of medical police, besides general observations, the subjects of celibacy; cohabitation; contagion; marriage; pregnancy; parturient women; Cæsarean operation; afflictive punishments; and inoculation.—This description applies to the latest edition of Professor Mahon's work, published in 1811.

Six bulky volumes, including upwards of three thousand pages, constitute the valuable *treatise of M. Foderé.* It is not so much to be considered an enlarged re-publication of the edition of 1796, as a new and more elaborate production. M. Foderé divides his work into three parts, of nearly equal extent; the first treating of mixed subjects, and alike applicable to civil and criminal jurisprudence, and to medical police: the second exclusively to criminal jurisprudence; and the third to medical police. Two volumes are occupied by each of these parts.—

To the first part are prefixed a preface, and an introduction; in the last of these, M. Foderé exposes the utility, and gives a definition, of juridical medicine; succinctly traces the history of the science from its origin; details the qualifications essential to its practice; and finally considers the means whereby it may be improved, and the difficulties which oppose its improvement. The different ages of human life, puberty, minority, majority; personal identity and resemblance; relative and absolute duration of life; grounds of prohibition, mental derangement, suicide, the deaf and dumb, somnambulism, intoxication, qualifications of witnesses and testators; marriage and divorce; pregnancy, genuine and spurious; parturition and the signs denoting the death of the fœtus; paternity and filiation; premature and procrastinated birth, monstrosities, fœtuses of dubious sex; survivorship; signs of the certainty of death, apparent death, treatment of different species of asphyxia; certificates of exemption and diseases which exempt; and feigned, concealed, and imputed diseases, are treated of in succession.

The second part, commencing at the third volume, includes, in their respective order, chapters on the juridical inspection of bodies, and the distinction of assassination from suicide; wounds; poisons; seduction, defloration, and rape; abortion, concealment, and substitution of the offspring, and infanticide.

In the third and last part, with which the fifth volume opens, are successively discussed, the preservation of the human species and means of remedying its physical degeneracy; contagious, hereditary, and epidemic diseases; medical police of cities, &c. in respect to aliment, arts, and manufactures; military and naval Hygiène; and, lastly, the medical police of hospitals and prisons.—Of the erudition and ability with which this vast and comprehensive plan has been executed by M. Foderé, few among our readers will require to be informed. His work, moreover, is copiously illustrated by records of the practice and decisions of the judicial courts of the continent upon almost every question which can become the subject of legislative research. Like most other French writers, he does not always escape the vice of needless prolixity and minuteness. In several instances, he complains loudly, and not without reason, that Mahon, or his Editor, has copied passages, and even chapters, from his first edition, without the slightest acknowledgment. Upon the whole, we regard the production of Foderé as yet unrivalled in its peculiar department of science; and gratefully acknowledge the assistance which, in the composition of the present article, as on many other occasions, we have derived from the labours of this learned and enlightened foreigner.

England, long celebrated for the publicity of her judicial proceedings, and for the institution of trial by jury, is far behind her neighbours in the culture of medical jurisprudence. Whether this neglect originate from the simplicity of our criminal code, or may be referred to an inherent peculiarity of national character, it would, perhaps, be difficult to determine. The fact, however, is as unquestionable as much to be lamented. It was not until the year 1803, that, on the presentation of a memorial urging the importance of such an establishment to the Patrons of the University, by the venerable Dr. Duncan a Professorship of Medical Jurisprudence was founded in the Edinburgh school. Dr. Duncan, junior, then elected to the chair, at present fills it with reputation to himself and advantage to the cause of public instruction. No other Professorship of this kind has yet been established in the British dominions.

Our catalogue of English works upon medical jurisprudence is, as may be supposed, exceedingly meagre.

With the exception of the very imperfect productions of Drs. Farr (1) and Bartley, (2) nothing, possessing even the semblance of a system of juridical medicine, has appeared in this island till very recently. The valuable Treatise on Medical Ethics, by Dr. Percival, of Manchester, cannot be said to come under this designation. Dr. Male's Epitome, published in the earlier part of the year just concluded, is the only work of which it now remains for us to speak. Under the head of Medical Police, we had nearly forgotten to mention an indifferent treatise, by Dr. Roberton, (3) which some years since made its appearance.

Dr. *Male*, in his *Epitome*, has adopted no particular system or arrangement. After a very sensible and well-written preface, he treats, in succession, of Poisons, animal, vegetable, mineral, and aërial; Wounds and Contusions; Infanticide; Pregnancy; Abortion and concealed Birth; Pretended Delivery; Rape; Hanging and Strangulation; Drowning; Dangerous Inebriety; Insanity; Pretended and imputed Diseases; Apparent Death; Impotence; and, lastly, Hermaphrodites. But all these articles are written in too slight and concise a manner to prove extensively useful to that class of men who stand most in need of instruction; and whose ignorance in every thing that regards juridical medicine we have daily fresh occasion to notice and deplore. This is the more to be regretted, as, from the clear and judicious manner in which Dr. Male writes, we feel convinced that he is deeply versed in his subject; and that few are better qualified than himself for the honourable task of filling this blank on the chart of English medical literature. We shall not here attempt an analysis of the work. The reader, on turning to the series of articles upon juridical medicine, which we have commenced in another department of our Journal, will immediately descry the motives of this deviation from the accustomed plan of our critical labours.

Another more systematic and comprehensive publication upon medical jurisprudence has recently been an-

---

(1) Elements of Medical Jurisprudence, &c. 12mo. London, 1788. (Third edition, 1815.)

(2) Treatise on Forensic Medicine, or Medical Jurisprudence. 12mo. Bristol, 1815.—For an account of this "meagre production," see New Med. and Phys. Journal, vol. x.

(3) Treatise on Medical Police, &c. vol. ii. 8vo. 1808.

nounced by a British writer. (1) This augurs well. We rejoice to see a spirit of emulation and inquiry going forth on this important but long-neglected branch of knowledge. We hope that the time is fast approaching, when every medical school in the British dominions, emulating the laudable example of the University of Edinburgh, will boast a Professor of Medical Jurisprudence; when every candidate for the honours of surgery or medicine will be required, ere he attain them, to evince a competent acquaintance with the general doctrines and principles of the science. We cannot recall, without pain and humiliation, the many flagrant examples of ignorance and apathy displayed by our medical practitioners, on questions involving the life and character of a human being. We cannot reflect, without shuddering, on the crowd of victims who might have been rescued from a scaffold; on the numerous reputable families whose peace and honour might have been saved from an irreparable wound by the zealous exertions of an enlightened professional evidence. Let him, who doubts the correctness of these observations, revert to the voluminous records of the English criminal courts — let him sit down, and dispassionately peruse the trial of the unfortunate Elizabeth Fenning (2)

If, among our readers, there be a young man destined for the medical profession, we exhort him, as he regards his future interests and reputation, as he values that sense of conscious integrity which will form the sweetest and least perishable reward of a life of solicitude and labour, sedulously to cultivate juridical medicine. It is to facilitate his progress in this little frequented path of science that we have principally been induced to trace a rude sketch of its history and literature. If self-interest be his ruling passion, let him reflect how many of his predecessors have been suddenly raised to eminence from obscurity — how many irreparably ruined and disgraced in the public opinion, by the development of previously unfolded talent, or the exposure of unsuspected ignorance, in a judicial examination. But if he possess a mind of more delicate structure and exalted views, we would, on the

---

(1) The name of the gentleman has escaped our recollection. Whoever he be, we earnestly exhort him to persevere in his laudable enterprize. REV.

(2) See the very interesting Report, published in 1815, relative to this extraordinary trial.

one side, represent to him the peace and conscious dignity with which he will retire from the successful exercise of his juridical knowledge in the cause of injured innocence; on the other, we would depict, in strongest colours, the remorse which cannot fail, sooner or later, to embitter his repose, if, through professional incapacity or neglect, or the influence of blind prejudices, he have been instrumental in destroying the character or life of a fellow creature, unfortunately implicated in his evidence and decisions.

To those who are well versed in the Latin and continental languages (and be it remembered that all medical education without this knowledge is defective), we have exposed an inexhaustible mine of literature in medical jurisprudence. They to whom these stores are inaccessible, will do well to make themselves thoroughly acquainted with the Epitome of Dr. Male; the writings of Drs. Parr,(1) Johnstone,(2) and Hunter; (3) and translations from Mahon (4) and Orfila, (5) on various branches of juridical research. Many valuable observations on these subjects, as well in the shape of review as of original contribution, enrich the pages of our highly respected contemporary, the *Edinburgh Medical and Surgical Journal.* As last and least of all, we may probably be allowed to recommend to the English *student* the series of articles now publishing, and expressly destined for his use, in the Selection Department of our own Journal.

In the difficulties attendant on the execution of the task which we are now concluding, some apology will perhaps be found for its numerous imperfections. We have no wish to exaggerate the one, or conceal the other: content with the humble merit of leading the young and inexperienced to a novel path of study; and only anxious to incite, both by precept aud example, the more mature practitioner to the culture of a science, the ignorance of which is not more derogatory to his own character than noxious in its operation upon the individual interests of the human heart, and on the welfare of human institutions.

---

(1) London Medical Dictionary, article Medicina Forensis.

(2) Essay on Mineral Poisons. 8vo. 1795. — Essay on Madness.

(3) On the Uncertainty of the Signs of Murder in the Case of Bastard Children. 8vo. 1812.

(4) Essay on the Signs of Murder in new-born Children, from the French of Mahon, by Johnson. Lancaster, 1813.

(5) A General System of Toxicology, &c. from the French of Orfila. See the Review Department at the close of our First, and commencement of our Second Volume.

